# Potential ^18^F-RGD PET/CT and DCE-MRI Imaging-Based Biomarkers for Postoperative Survival Prediction Among Patients With Newly Diagnosed Glioblastoma Treated With Bevacizumab and Chemoradiotherapy

**DOI:** 10.3389/fonc.2022.848266

**Published:** 2022-08-26

**Authors:** Li Li, Ning Liu, Hui Zhang, Rongjie Tao, Shuqiang Zhao, Zhaoqiu Chen, Zheng Fu, Wanhu Li, Liang Xu, Yuhui Liu, Jinming Yu, Shuanghu Yuan

**Affiliations:** ^1^ Department of Radiation Oncology, Shandong Cancer Hospital and Institute, Shandong First Medical University and Shandong Academy of Medical Sciences, Jinan, China; ^2^ Department of Oncology, Linyi Cancer Hospital, Linyi, China; ^3^ Department of Neurosurgery, Shandong Cancer Hospital and Institute, Shandong First Medical University and Shandong Academy of Medical Sciences, Jinan, China; ^4^ Department of PET/CT Center, Shandong Cancer Hospital and Institute, Shandong First Medical University and Shandong Academy of Medical Sciences, Jinan, China; ^5^ Department of Radiology, Shandong Cancer Hospital and Institute, Shandong First Medical University and Shandong Academy of Medical Sciences, Jinan, China; ^6^ Department of Radiation Oncology and Shandong Provincial Key Laboratory of Radiation Oncology, Shandong Cancer Hospital and Institute, Shandong First Medical University and Shandong Academy of Medical Sciences, Jinan, China; ^7^ Research Unit of Radiation Oncology, Chinese Academy of Medical Sciences, Jinan, China; ^8^ The Affiliated Cancer Hospital of Zhengzhou University, Henan Cancer Hospital, Zhengzhou, China

**Keywords:** Glioblastoma, ^18^F-RGD PET/CT, DCE-MRI, bevacizumab, concurrent radiotherapy and temozolomide, PFS, OS

## Abstract

**Purpose:**

To investigate the ability of potential imaging biomarkers based on ^18^F-AlF-NOTA-PRGD2 positron emission tomography/computed tomography (^18^F-RGD PET/CT) and dynamic contrast-enhanced magnetic resonance imaging (DCE-MRI) imaging to predict the response to bevacizumab combined with conventional therapy in postoperative newly diagnosed glioblastoma.

**Methods:**

Twenty patients with newly diagnosed with glioblastoma after surgery were prospectively enrolled to receive bevacizumab plus conventional concurrent radiotherapy and temozolomide (CCRT). ^18^F-RGD PET/CT and DCE-MRI were performed at baseline, week 3, and week 10 for each patient. Statistical methods included the analysis of variance (ANOVA), Kaplan–Meier method and Cox proportional hazard analysis.

**Results:**

All patients completed CCRT plus bevacizumab therapy without interruption. The median follow-up time was 33.9 months (95% confidence interval [CI], 28.3-39.5 months). The median progression-free survival (PFS) and overall survival (OS) was 9.66 months (95% CI, 6.20-13.12 months) and 15.89 months (95% CI, 13.89-17.78), respectively. Treatment was generally well tolerated, and there were no Treatment emergent adverse events (TEAEs) with a toxicity grade equal to or exceeding 3 or that led to termination of treatment or patient death.Over the treatment interval of bevacizumab therapy from week 3 to week 10, patients with a large decrease of SUVmean was associated with a better PFS with a hazard ratio (HR) of 6.562, 95% CI (1.318-32.667), p=0.022. According to Kaplan-Meier analysis, patients with a decrease in the SUVmean of more than 0.115 on ^18^F-RGD PET/CT had a longer PFS than those with a decrease in the SUVmean of 0.115 or less (12.25 months vs.7.46 months, p=0.009). For OS, only a small decrease of Ktrans was also found to have certain prognostic value (HR=0.986, 95% CI (0.975-0.998), p=0.023). Patients with a decrease in Ktrans larger than 37.03 (min-1) on DCE-MRI had worse OS than those with a decrease in Ktrans of 37.03 (min-1) or less (15.93 months vs. 26.42 months, p=0.044).

**Conclusion:**

^18^F-RGD PET/CT and DCE-MRI may be valuable in evaluating the response of glioblastoma to treatment with the combination of bevacizumab and CCRT, with a greater decrease in SUV_mean_ predicting better PFS as well as a small decrease in K^trans^ predicting improved OS.

## Introduction

Glioblastoma is the most common and devastating type of primary intracranial tumor (1). According to the 2021 statistical report of the Central Brain Tumor Registry of the United States (CBTRUS), glioblastoma accounts for 49.1% of all malignant brain and central nervous system (CNS) tumors and 58.4% of gliomas (2). Because vascular endothelial growth factor (VEGF) and VEGF receptor (VEGFR) are up-regulated in glioblastoma, antiangiogenic drugs targeting VEGF ligand have been suggested and introduced into the treatment regime with radiotherapy plus concurrent or adjuvant temozolomide (3, 4). These antiangiogenic drugs may inhibit angiogenesis primarily through destruction of existing tumor vasculature, normalization of surviving vessels, and inhibition of new and relapsing tumor blood vessel growth (5–7). Bevacizumab, a humanized monoclonal antibody targeted to the VEGFA ligand, was approved for the treatment of recurrent glioblastoma rather than newly diagnosed glioblastomas by the Food and Drug Administration (8). A series of prospective clinical trials had yielded some results, patients treated with bevacizumab combined with conventional concurrent radiotherapy and temozolomide (CCRT) revealed a median overall survival (OS) ranging from 15.7-19.6 months and median progression-free survival (PFS) 8.4-13.6 months versus those only received CCRT who had a median OS ranging from 14.6-21.1 months and PFS 4.3-9.4 months (9, 10). The PFS rather than OS of patients with newly diagnosed glioblastoma treated with bevacizumab combined with CCRT was significantly improved. However, the OS has been found to be heterogeneous, with a considerable number of patients still benefiting significantly from this combination therapy.

The ability to accurately screen this portion of the population benefiting from PFS and OS could provide a breakthrough in the treatment of newly diagnosed glioblastoma patients. It is well-established that glioblastoma patients with promoter methylation of the DNA repair enzyme O6-methylguanine-DNA methyltransferase (MGMT) derive more benefit from treatment with temozolomide (11). However, the prediction of the response to antiangiogenic therapy has always been a complicated problem. VEGFA is the major mediator promoting tumor-induced angiogenesis (4). Its predictive value for the response to antiangiogenic therapy has been extensively studied, but inconsistent prediction results have limited its widespread application in clinical practice (12–15). There remains no appropriate parameter for predicting the response of glioblastoma to bevacizumab as precisely as epidermal growth factor receptor (EGFR) mutation predicts the response to tyrosine kinase inhibitor (TKI) treatment (16, 17).

As bevacizumab achieves an anti-tumor effect mainly *via* changing the density of microvessels and decrease vascular permeability, blood flow, and the degree of oxygenation in the local microenvironment (18), it may be a more rational direction to search for prediction parameters through dynamic imaging examination to solve the current dilemma. Arginine-glycine–aspartic acid (RGD) is the most important integrin for angiogenesis, because it specially binds to the integrin alpha V beta3 (αvβ3), which is highly expressed in new tumor vessels (19, 20). In theory, RGD positron emission tomography (PET) can provide direct imaging of angiogenesis, and ^18^F-Galacto-RGD uptake was shown to be significantly correlated with αvβ3 integrin staining intensity in glioblastoma lesions (21). Recently, the novel one-step method for preparing the integrin αvβ3-targeting PET probe ^18^F-AlF-NOTA-PRGD2 has made tracer preparation more convenient, which then increases the likelihood that this imaging method can be popularized in clinical practice (22). Quantitative permeability parameters such as the volume transfer constant (K^trans^) on dynamic contrast-enhanced magnetic resonance imaging (DCE-MRI) may also reflect the physiologic characteristics of the microvasculature, permeability, and angiogenesis (23, 24).

Therefore, we registered and performed the present prospective study. ^18^F-AlF-NOTA-PRGD2 and DCE-MRI were completed to identify potentially effective biomarkers for the prognosis of newly diagnosed glioblastoma after surgery treated with the combination of bevacizumab with CCRT.

## Materials and Methods

This was an open-label, single-arm, phase IV clinical trial involving patients with newly diagnosed glioblastoma (World Health Organization [WHO], Grade IV) that was confirmed histopathologically after surgery. This trial was approved by the ethics committee of Shandong Cancer Hospital and Institute (number NCT01939574, ID ML28676).

### Patient Selection

Patients histologically proven glioblastoma were prospectively enrolled in the study. The inclusion criteria were: residual tumor volume less than 25% of the preoperative tumor volume; postoperative Karnofsky Performance status (KPS) score no less than 70; no postoperative infection or other complications before initiation of CCRT; adequate organ function; and age 18 years or older. The exclusion criteria included: recurrent or multifocal malignant glioma, prior treatment with chemotherapy or radiosensitizers for cancers of the head and neck region, history of myocardial infarction or stroke within 6 months, inability to undergo MRI or PET/computed tomography (CT) imaging, and pregnancy. All patients were informed of the study procedure and then provided specific informed consent prior to enrollment.

### Treatment Plan

All patients received CCRT plus bevacizumab and adjuvant temozolomide plus bevacizumab (starting >3 weeks and ≤5 weeks after surgical treatment). Patients were treated with post-radiation bevacizumab and temozolomide for 6 cycles unless there was evidence of tumor progression or treatment-related toxicity or if a patient requested to withdraw from the study.

#### Concurrent CCRT Plus Bevacizumab Therapy

Radiotherapy (RT): For both intensity-modulated radiotherapy (IMRT) and 3D-conformal radiotherapy (3D-CRT) plans, 2 Gy was given once daily, 5 days per week for a total of 60 Gy over 6 weeks.

Temozolomide: Temozolomide was administered continuously from day 1 to the last day of radiation at a daily oral dose of 75 mg/m^2^ for a maximum of 49 days.

Bevacizumab: Bevacizumab was administered intravenously on days 1 and 15 of each 28-day cycle, at the beginning of the 4th week of radiation. The dose was 10 mg/kg.

#### Adjuvant Therapy

Temozolomide: Temozolomide was administered orally once per day for 5 consecutive days (days 1–5) of a 28-day cycle. The starting dose for the first cycle was 150 mg/m^2^/day, with a single dose escalation to 200 mg/m^2^/day in subsequent cycles if no treatment-related adverse events exceeding grade 2 were noted.

Bevacizumab: Bevacizumab was administered intravenously on days 1 and 15 of each 28-day cycle. The dose was 10 mg/kg of actual body weight.

### Imaging Processing Protocols

**Figure 1:**
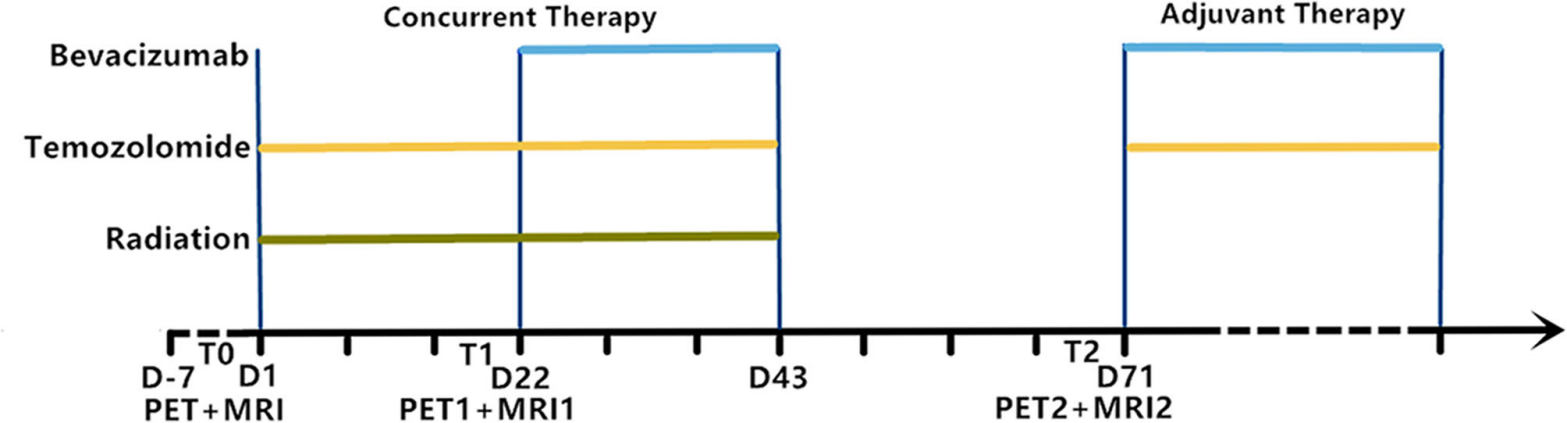
Experimental timeline showing relative timing of radiation, temozolomide and bevacizumab treatment as well as ^18^F-RGD PET and DCE-MRI image acquisition. T_0_, baseline; T_1_, week 3; T_2_, week 10; D, days.

Patients underwent ^18^F-RGD PET/CT and DCE-MRI at four time points: T_0_ (baseline, corresponding to 0–5 days before the initiation of CCRT); T_1_ (the third week, corresponding to 0–3 days before the commencement of bevacizumab therapy); T_2_ (the tenth week, corresponding to 7 weeks after the commencement of bevacizumab therapy). The examinations on T_m_-point were optional for patients (**Figure 1**).

#### ^18^F-RGD PET/CT Protocol

All PET scans were obtained using a dedicated PET/CT scanner (GEMINI TF Big Bore; Philips Healthcare). ^18^F-RGD was synthesized according to a previously reported procedure (22) and injected intravenously at a mean dose of 1.89 ± 0.37 MBq/kg. Patients underwent PET scans covering the whole head after intravenous radiopharmaceutical administration for approximate 60 min. First, low-dose CT was applied over the same region of interest for attenuation correction. These images were viewed on a Xeleris workstation (GE Healthcare).

#### DCE-MRI Protocol

MRI scans were performed using a 3.0-Tesla MRI system (SIEMENS). The protocols consisted of axial proton density, T1- and T2-weighted fast spin-echo images, and fluid-attenuated inversion recovery (FLAIR) images. After T1-weighted imaging (repetition time [TR]/echo time [TE], 500/12.1 ms, slice thickness, 5 mm, 16 axial slices; matrix, 192×256; number of incentives [NEX], 1). This was followed by a DCE acquisition series (TR, 150 ms; TE, 4 ms; FOV, 240 mm×240 mm; slice thickness, 5 mm; and matrix, 128×128) with a flip angle of 30°, which consisted of 60 measurements with temporal spacing of 90 s. Meanwhile, 0.1 mmol/kg body weight of gadopentetate dimeglumine contrast agent Gd-diethylenetriamine pentaacetic acid-bismethylamide (Gd-DTPA) was administered intravenously at a rate of 3 ml/s.

### Data Processing and Analysis

^18^F-RGD PET and DCE-MRI images were analyzed separately for each patient by an experienced neuroradiologist. The information of the target resection in surgery provided references to sketch the regions of interests (ROIs) in PET and MRI.

#### ^18^F-RGD PET/CT Processing

The physiological ^18^F-RGD uptake was obtained in the ROIs involving the entire contralateral hemisphere at the level of the centrum semiovale (25). The threshold with 1.5 of the mean standard uptake value (SUV_mean_) of the normal hemispheric background was used in cases of the tumor involving the striatum (26). Maximum and mean standard uptake values (SUV_max_ and SUV_mean_) within the volume were determined, and the corresponding tumor-to-normal brain ratio (TNR_max_ and TNR_mean_) were also analyzed.

#### DCE-MRI Processing

The permeability parameters of DCE-MRI were calculated by off-line Pride tools provided by Philips Medical System, which was based on the pharmacokinetic model of Tofts (27). Post-processing included motion correction of pixels from dynamic images, T1 mapping and registration of pixels on a T1 map, arterial input function estimation, and pharmacokinetic modeling. An arterial input function (AIF) was generated from a chosen section of the internal carotid artery. Three ROIs were drawn at the maximal enhancing portion of the remaining tumor tissue on contrast-enhanced T1 weighted images. The ROIs were duplicated by simultaneous observation on axial post-contrast T1-weighted MRI, and corresponding permeability maps were generated automatically by Pride tools. Normal microvessels as well as cystic, necrotic, and hemorrhagic regions within the ROIs were avoided during ROI selection. Then the mean values of permeability parameters K^trans^, reflux constant (Kep), extravascular extracellular space volume per unit volume of tissue (Ve), and blood plasma volume per unit volume of tissue (Vp) were evaluated.

### Endpoints

The primary endpoint was PFS defined as the time from the start of therapy to disease progression or death due to any cause or censoring when the last patient finished the 12 months of follow-up, whichever occurred first. Progression was evaluated according to the updated Response Assessment in Neuro-Oncology (RANO) criteria (28), based on MRI performed at baseline, week 3, week 10, at the end of treatment, and every 2 months during the follow-up period. Treatment emergent adverse events (TEAEs) were also recorded according to the Introductory Guide MedDRA Version 21.1 (29).

### Statistical Analysis

Statistical analysis was performed using the commercial software SPSS 20.0 (SPSS, Inc.). The biomarkers were evaluated based on values at baseline and differences from baseline to week 10, and week 3 to week 10 in the context of survival. Analysis of variance (ANOVA) was used to evaluate the differences among biomarkers at separate time points. Patients were classified into two groups based on the median value. The prognostic significance of different groups was evaluated using Cox regression analyses and compared by the Kaplan–Meier method. Statistical significance was set at a p value less than 0.05.

## Results

### Study Population

Twenty patients were prospectively enrolled from November 20, 2013 to June 16, 2015. Among the study population, the median age was 50 years and the median KPS score was 80 (range, 70–90). Additional patient characteristics are listed in **Table 1**. All patients completed concurrent CCRT plus bevacizumab therapy without interruption. Patient No. 16 stopped therapy with bevacizumab due to hemoptysis during adjuvant therapy.

**Table 1:** Patients’ characteristics.

Characters	n (%)
**Age (years)**	
≤50	11 (55%)
>50	9(45%)
**Gender**	
Male	14 (70%)
Female	6 (30%)
**Karnofsky Performance status** 80 (range, 70–90)	20 (100%)
**Smoking status**	
Never-smoker	14 (70%)
Former-smoker	4 (25%)
Current-smoker	1 (5%)
**Location**	
Frontal lobe	3 (15%)
Temporal lobe	5 (25%)
Parietal lobe	1 (5%)
Occipital lobe	3 (15%)
Thalamus	1 (5%)
Others	7 (35%)
**WHO grade**	IV
**Histology**	Glioblastoma
**Surgery**	Partial section
**Radiotherapy**	60Gy/30fractions/6weeks

### Survival

The median follow-up time was 33.9 months (95% confidence interval [CI], 28.3-39.5 months). Nineteen patients experienced progressive disease as a primary endpoint. The median PFS was 9.66 months (95% CI, 6.20-13.12 months), the 25% and 75% PFS durations were 12.32 and 7.0 months, respectively. The median overall survival (OS) was 15.89 months (95% CI, 13.89-17.78), the 25% and 75% OS were 9.69 and 26.12 months, respectively. At the end of follow-up, 4 patients (20%) were still alive with a median OS of 35.4 months (range 31.6-41.9).

### Treatment Toxicity and Treatment-Dependent Parameter Alterations

Treatment was generally well tolerated, and the side effects followed the established toxicity profile of each drug. Among the 20 patients, five patients developed TEAEs related to the bevacizumab, accounting for 25% of all cases. In the present study, there were no TEAEs with a toxicity grade equal to or exceeding 3 or that led to termination of treatment or patient death (**Table 2**).

**Table 2:** TEAEs experienced among the study population.

TEAEs	N=20, Grade, n (%)				
	Grade 1	Grade 2	Grade 3	Grade 4	Grade 5
**Respiratory, thoracic and mediastinal disorders**	0	1 (5.0)	0	0	0
Hemoptysis	0	1 (5.0)	0	0	0
**Immune system disorders**	1 (5.0)	0	0	0	0
Drug hypersensitivity reaction	1 (5.0)	0	0	0	0
**Skin and subcutaneous tissue disorders**	0	1 (5.0)	0	0	0
Erythema	0	1 (5.0)	0	0	0
Pruritus	0	1 (5.0)	0	0	0
**General disorders and administration site conditions**	2 (10.0)	1 (5.0)	0	0	0
Fatigue	2 (10.0)	0	0	0	0
Chest pain	0	1 (5.0)	0	0	0
**Gastrointestinal disorders**	0	1 (5.0)	0	0	0
Nausea	0	1 (5.0)	0	0	0
Vomiting	0	1 (5.0)	0	0	0
Gastrointestinal disorders	1 (5.0)	0	0	0	0
Gastroesophageal reflux disease	0	1 (5.0)	0	0	0

The treatment-dependent alterations of ^18^F-RGD PET/CT and DCE-MRI parameters are presented in **Table 3**. The correlations between biomarkers derived from DCE-MRI and ^18^F-RGD PET/CT were also analyzed in **Table 4**, it revealed that baseline ^18^F-RGD PET/CT parameters (SUV_mean_, TNT_mean_, SUV_max_) were positively correlated with DCE-MRI parameters K^trans^ and Vp (SUV_mean_ and K^trans^: r=0.747, p<0.001; TNT_mean_ and K^trans^: r=0.445, p=0.049; SUV_max_ and K^trans^: r=0.576, p=0.008; SUV_mean_ and Vp: r=0.483, p=0.031).

**Table 3:** Treatment-dependent alterations of 18F-RGD PET and DCE-MRI parameters.

Parameters		Baseline	Week 3	Week 10	p
DCE-MRI	Kep(min^-1^)	453.57 ± 263.55	521.48 ± 436.42	280.92 ± 71.78	0.19
	K^trans^(min^-1^)	183.34 ± 105.20	155.77 ± 65.58	97.53 ± 46.97	0.04
	Ve	462.09 ± 219.04	371.06 ± 189.22	348.93 ± 202.46	0.398
	Vp	116.69 ± 47.60	78.05 ± 39.58	48.90 ± 24.81	0.001
^18^F-RGD PET/CT	SUVmean	0.59 ± 0.21	0.54 ± 0.15	0.43 ± 0.22	0.106
	TNTmean	8.22 ± 3.96	10.02 ± 6.39	6.05 ± 2.82	0.089
	SUVmax	1.68 ± 0.71	1.56 ± 0.86	1.04 ± 0.62	0.058
	TNTmax	22.97 ± 11.12	28.18 ± 19.55	13.64 ± 7.32	0.025

Kep, reflux constant; K^trans^, the volume transfer constant; Ve, extravascular extracellular space volume per unit volume of tissue; Vp, blood plasma volume per unit volume of tissue; SUV_max_, Maximum standard uptake value; SUV_mean_, mean standard uptake value; TNR_max_ and TNR_mean_, SUV_max_ and SUV_mean_ of tumor-to-normal brain ratio.

**Table 4:** Spearman correlation coefficient between biomarkers derived from DCE-MRI and ^18^F-RGD PET/CT.

Time Point	parameters	Kep		K^trans^		Ve		Vp	
**Baseline**	SUV_mean_	r=0.368	p=0.110	*r=0.747*	*p=0.000*	r=0.360	p=0.119	*r=0.483*	*p=0.031*
	TNT_mean_	r=-0.005	p=0.984	*r=0.445*	*p=0.049*	r=0.438	p=0.054	r=0.402	p=0.079
	SUV_max_	r=0.262	p=0.265	*r=0.576*	*p=0.008*	r=0.315	p=0.176	r=0.381	p=0.097
	TNT_max_	r=0.008	p=0.974	r=0.277	p=0.237	r=0.255	p=0.277	r=0.220	p=0.350
**Week 3**	SUV_mean_	r=-0.452	p=0.069	r=-0.354	p=0.163	r=0.074	p=0.779	r=0.465	p=0.060
	TNT_mean_	r=-0.270	p=0.295	r=0.079	p=0.764	r=0.285	p=0.268	r=0.238	p=0.357
	SUV_max_	r=-0.317	p=0.215	r=-0.317	p=0.215	r=-0.002	p=0.994	r=0.278	p=0.280
	TNT_max_	r=-0.276	p=0.284	r=-0.072	p=0.783	r=0.140	p=0.592	r=0.237	p=0.360
**Week 10**	SUV_mean_	r=-0.095	p=0.782	r=0.188	p=0.579	r=0.230	p=0.497	r=-0.134	p=0.695
	TNT_mean_	r=-0.018	p=0.958	r=0.283	p=0.399	r=0.313	p=0.348	r=0.050	p=0.884
	SUV_max_	r=-0.200	p=0.555	r=0.092	p=0.788	r=0.243	p=0.471	r=-0.279	p=0.406
	TNT_max_	r=-0.165	p=0.629	r=0.202	p=0.552	r=0.336	p=0.313	r=-0.087	p=0.800

Ktrans, the volume transfer constant; Kep, reflux constant; Ve, extravascular extracellular space volume per unit volume of tissue; Vp, blood plasma volume per unit volume of tissue; SUVmax, Maximum standard uptake value; SUVmean, mean standard uptake value; TNRmax and TNRmean, SUVmax and SUVmean of tumor-to-normal brain ratio.

### Associations Between Imaging Biomarkers and PFS

The results of univariate analysis to identify effective biomarkers (at baseline, between baseline and week 10, and between weeks 3 and 10) for survival are summarized in **Table 5**. Among the eight variables tested, baseline value or changes from baseline to week 10 on ^18^F-RGD PET/CT were found not to be predictive of PFS. However, over the course of the first cycle of bevacizumab therapy from week 3 to week 10, a large decrease of SUV_mean_ was associated with a better PFS with a hazard ratio (HR) of 6.562, 95% CI (1.318-32.667), p=0.022.

**Table 5:** Univariate Cox regression analyses of factors predictive of survival.

Factor	Progression-free Survival						Overall Survival					
	Value at baseline		Change from baseline to week 10		Change from week 3 to week 10		Value at baseline		Change from baseline to week 10		Change from week 3 to week 10		
	HR (95.0% CI)	p	HR (95.0% CI)	p	HR (95.0% CI)	p	HR (95.0% CI)	p	HR (95.0% CI)	p	HR (95.0% CI)	p	
Gender	*7.252 (1.567-33.56)*	*0.011*	–	–	–	–	2.228 (0.757-6.552)	0.146	–	–	–	–	
Age	0.973 (0.928-1.020)	0.262	–	–	–	–	1.032 (0.958-1.113)	0.405	–	–	–	–	
KPS	1.026 (0.952-1.105)	0.505	–	–	–	–	1.593 (0.512-4.955)	0.421	–	–	–	–	
Smoking status	reference	0.474	–	–	–	–	–	0.239	–	–	–	–	
	1.135 (0.143-9.009)	0.905	–	–	–	–	0.298 (0.034-2.588)	0.273	–	–	–	–	
	2.173 (0.249-18.927)	0.482	–	–	–	–	0.737 (0.074-6.327)	0.737	–	–	–	–	
TV	0.361 (0.123-1.059)	0.063	1.065 (0.339-3.342)	0.914	0.686 (0.204-2.303)	0.542	0.772 (0.227-2.154)	0.621	0.350 (0.094-1.308)	0.119	0.842 (0.239-2.960)	0.788	
Kep	1.166 (0.447-3.038)	0.753	1.452 (0.381-5.533)	0.585	1.132 (0.299-4.289)	0.856	0.414 (0.148-1.162)	0.094	0.478 (0.113-2.015)	0.315	1.676 (0.383-7.328)	0.493	
K^trans^	0.581 (0.214-1.578)	0.287	0.512 (0.135-1.936)	0.324	1.202 (0.318-4.538)	0.786	1.859 (0.687-5.031)	0.222	0.821 (0.200-3.365)	*0.784*	*0.986 (0.975-0.998)*	*0.023*	
Ve	0.948 (0.364-2.466)	0.912	0.459 (0.122-1.729)	0.25	0.815 (0.217-3.062)	0.762	0.998 (0.996-1.000)	0.108	0.779 (0.190-3.193)	729	0.209 (0.039-1.110)	0.066	
Vp	0.813 (0.320-2.067)	0.664	1.556 (0.413-5.863)	0.514	1.761 (0.462-6.706)	0.407	0.877 (0.325-2.365)	0.795	0.581 (0.140-2.405)	0.454	1.457 (0.347-6.119)	0.607	
SUV_mean_	0.691 (0.258-1.850)	0.462	0.711 (0.223-2.261)	0.563	*6.562 (1.318-32.667)*	*0.022*	1.787 (0.654-4.880)	0.257	1.201 (0.364-3.967)	0.764	1.839 (0.555-6.096)	0.319	
TNR_mean_	0.743 (0.278-1.984)	0.553	0.778 (0.243-2.497)	0.673	1.471 (0.462-4.686)	0.514	1.106 (0.414-2.959)	0.84	1.842 (0.556-6.104)	0.318	1.151 (0.349-3.797)	0.817	
SUV_max_	0.691 (0.258-1.850)	0.462	1.129 (0.356-3.583)	0.837	2.000 (0.627-6.383)	0.242	1.787 (0.654-4.880)	0.257	0.982 (0.297-3.243)	0.976	1.004 (0.303-3.323)	0.995	
TNR_max_	0.581 (0.214-1.578)	0.287	1.958 (0.619-6.193)	0.253	0.788 (0.247-2.517)	0.688	1.859 (0.687-5.031)	0.222	1.388 (0.420-4.588)	0.591	1.337 (0.401-4.453)	0.636	

KPS, Karnofsky Performance status; TV, Tumor Volume; Kep, reflux constant; K^trans^, the volume transfer constant; Ve, extravascular extracellular space volume per unit volume of tissue; Vp, blood plasma volume per unit volume of tissue; SUV_max_, Maximum standard uptake value; SUV_mean_, mean standard uptake value; TNR_max_ and TNR_mean_, SUV_max_ and SUV_mean_ of tumor-to-normal brain ratio.

**Figure 2:**
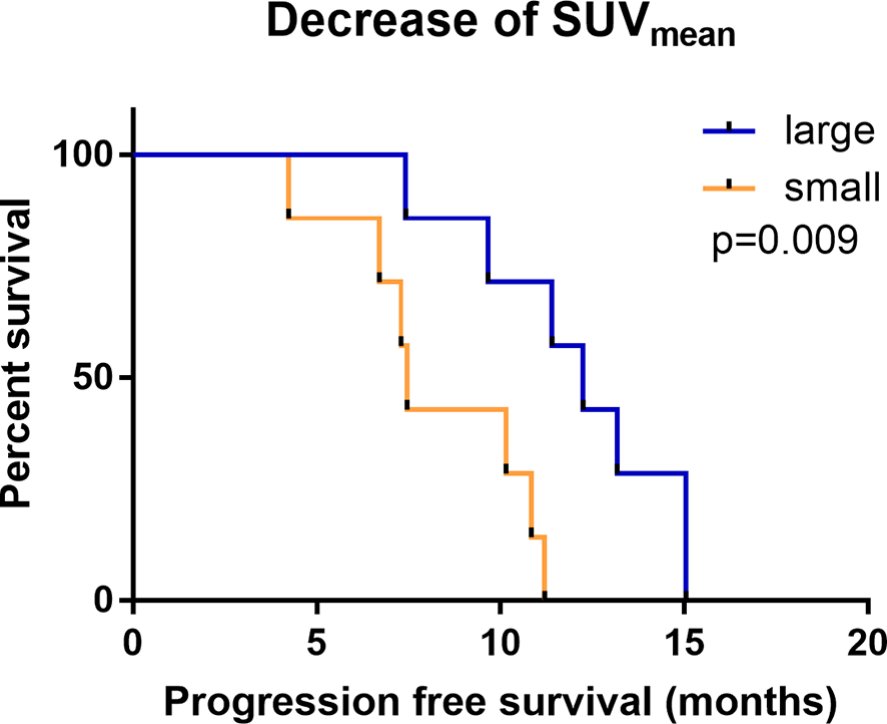
Kaplan-Meier analysis of PFS comparing groups with a large (red) and small (blue) change in the SUV_mean_.

**Figure 3:**
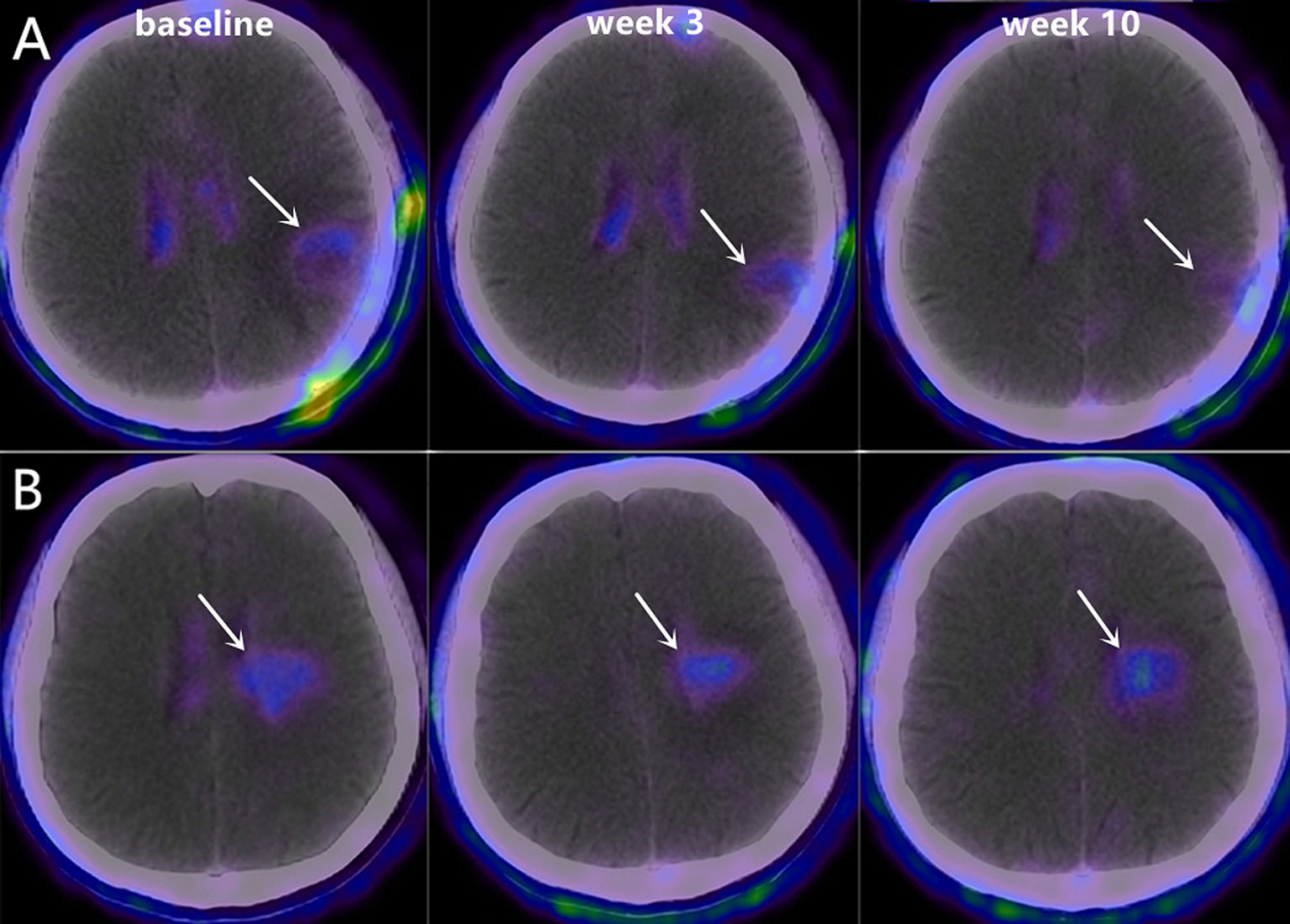
Representative ^18^F-RGD PET/CT scans at baseline, week 3 and week 10 in two patients with PFS of 9.65 months (**A**, Male, 48 years old, decrease in SUV_mean_=-0.45) and 7.45 months (**B**, Male, 62 years old, decrease in SUV_mean_=0.25).

According to Kaplan-Meier analysis, patients with a decrease in the SUV_mean_ of more than 0.115 on ^18^F-RGD PET/CT had a longer PFS than those with a decrease in the SUV_mean_ of 0.115 or less (12.25 months vs.7.46 months, p=0.009; **Figures 2**, **3**). The SUV_mean_ differed significantly between the groups with high and small decreases, and the decrease in the SUV_mean_ in patients who showed better PFS was greater than that in those who showed worse PFS (-0.279 ± 0.102 vs. 0.061 ± 0.123, p<0.001).

### Associations Between Imaging Biomarkers and OS

Over the treatment interval of bevacizumab therapy from week 3 to week 10, only a small decrease in K^trans^ was also found to have certain prognostic value (HR=0.986, 95% CI (0.975-0.998), p=0.023).

**Figure 4:**
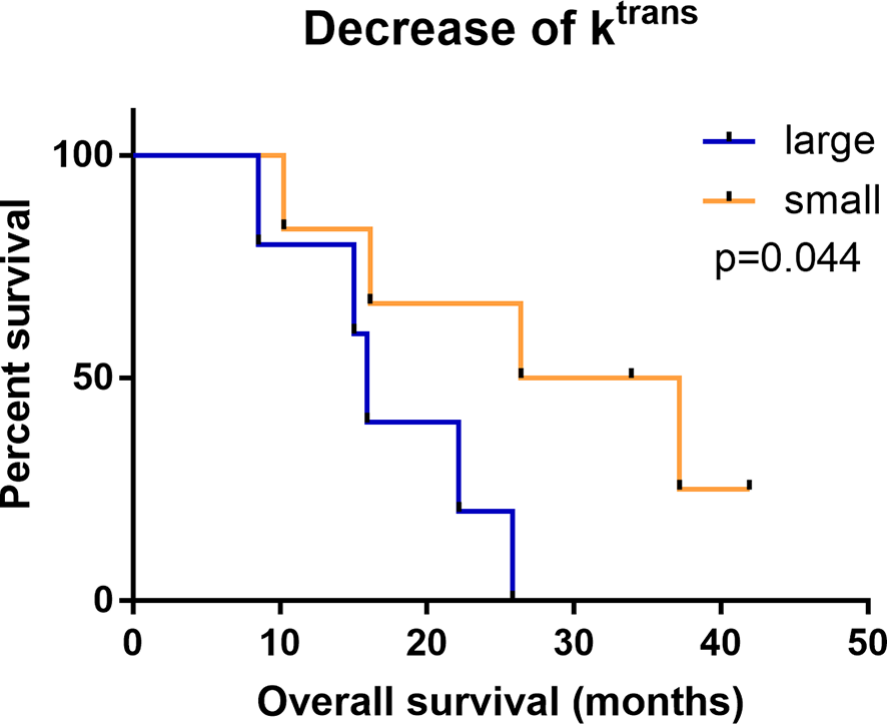
Kaplan-Meier analysis of OS comparing groups with a large (red) and small (blue) change in the K^trans^.

**Figure 5:**
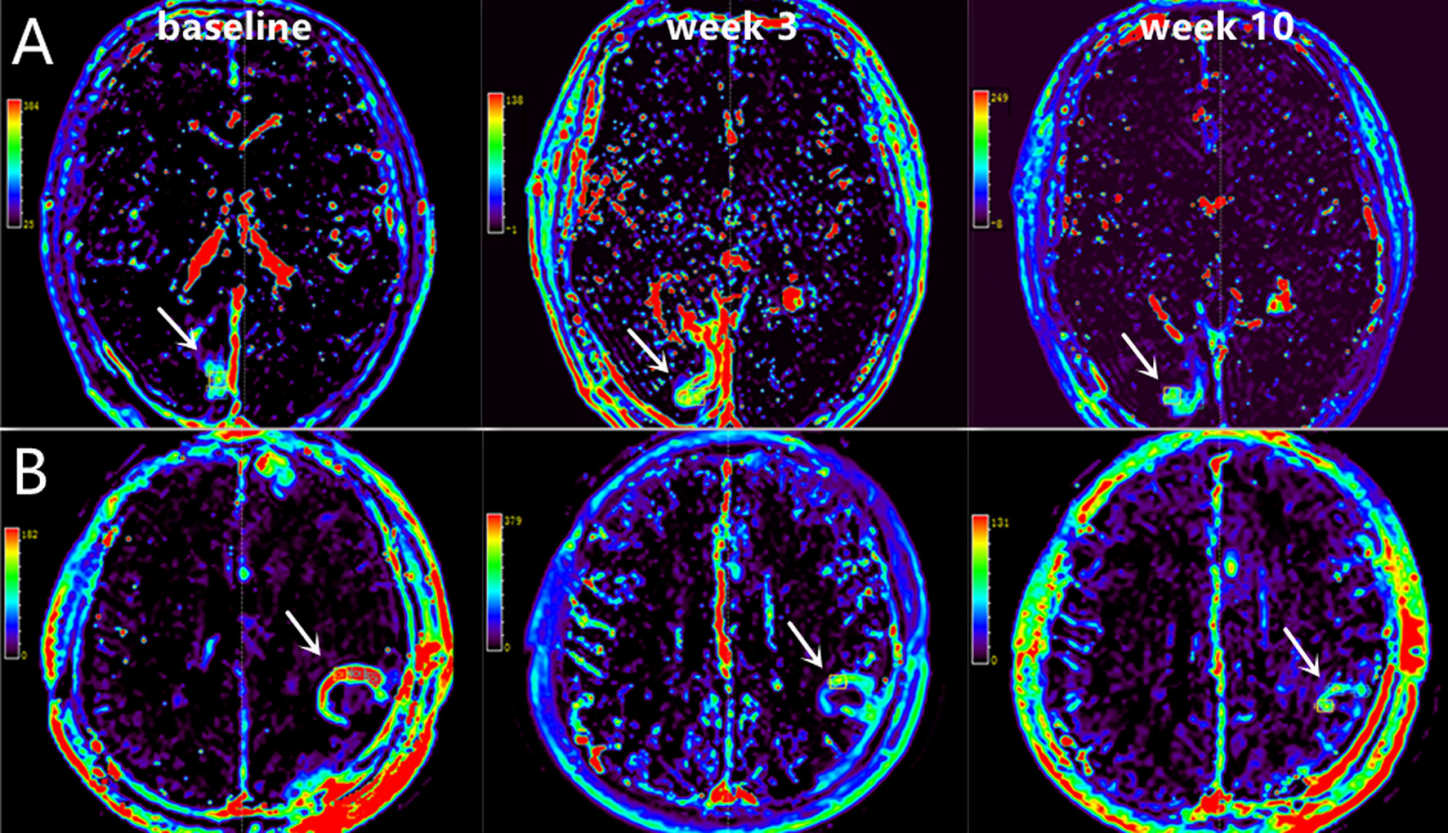
Representative DCE-MRI scans at baseline, week 3 and week 10 in two patients with OS of 41.92 months [**A**, Male, 53 years old, decrease in K^trans^=13.12 (min^-1^)] and 22.18 months [**B**, Male, 48 years old, decrease in K^trans^=-139.82 (min^-1^)].

With the cut-off value of -37.03 (min^-1^), the OS differed significantly between patients with large vs. small decrease in K^trans^ (large vs. small, 15.93 months vs. 26.42 months, p=0.044; **Figures 4**, **5**). The difference in the K^trans^ changes was statistically significant between patients who showed better OS and those who showed worse OS (10.2 ± 41.54 vs. -140.3 ± 50.88, p<0.001).

## Discussion

This prospective study examined the potential value of imaging biomarkers for assessing the prognosis of patients with postoperative newly diagnosed glioblastoma treated with bevacizumab combined with CCRT. A large decrease in the tumor SUV_mean_ on RGD-PET for PFS as well as small decrease in K^trans^ on DCE-MRI from week 3 to week 10 were favorable factors for improved survival, indicating that these imaging modalities offer early screening benefits for patients treated with combination therapy to better guide treatment planning.

Our exciting findings of the value of the new imaging markers for predicting survival following treatment with bevacizumab and CCRT were in accordance with the prior literature. Provost et al. reported that imaging *via* ^68^Ga-RGD PET/CT rather than ^18^F-FDG PET/CT revealed changes in the SUV of tumors following bevacizumab and/or temozolomide treatment in mice models bearing a U87MG tumor (30). In another study employing an bevacizumab-containing therapy in six cases of ovarian and cervical cancers, a large decrease in the RGD uptake was noted in patients with an early objective response (31). Eight glioblastoma patients received bevacizumab treatment and underwent pretreatment ^18^F-FPPRGD2 PET, and in these patients, a small decrease in ^18^F-FPPRGD2 uptake tended to indicate a poor prognosis (32). Xia et al. showed that a lower decrease in K^trans^ was associated with a better response to antiangiogenic treatment followed by chemoradiotherapy in 11 soft tissue sarcoma patients (33). However, these studies were limited by the use of only animal models, a small sample size, or a non-standard treatment mode. They mainly focused on monitoring rather than comparing survival to identify clinical characteristics associated with statistically significant survival benefits.

The newly diagnosed glioblastoma patients enrolled in this study postoperatively were receiving the standard combination treatment of bevacizumab with CCRT and underwent dynamic combined imaging examination of RGD PET and DCE-MRI. As described in previous studies, RGD PET served as an excellent imaging method for glioblastoma patients with a clear background, according to the passage of the tracer through the damaged blood-brain barrier within the tumor (22, 34). Patients with a larger decrease in the RGD SUV_mean_ from before to after bevacizumab treatment significantly showed a better PFS. Bevacizumab is a humanized monoclonal antibody targeting VEGFA. It limits tumor growth mainly by blocking the blood supply within the existing tumor vasculature, inhibiting the function of the tumor microvasculature as well as endothelial cell migration, thereby preventing regrowth over time (18, 35). The complexes formed by αvβ3 integrin and VEGFRs are considered to be the most important integrin interactions in angiogenesis, and the αvβ3-targeting RGD uptake values may reflect VEGF pathway activity (36–38). Therefore, the greater decrease in SUV_mean_ may represent stronger inhibition of tumor neovascularization and migration by bevacizumab to achieve superior antiangiogenic efficacy.

In addition, as shown in our study, K^trans^ showed a significant decrease during the period of combination therapy, and a small decrease in K^trans^ from week 3 to week 10 on DCE-MRI was predictive of improved OS. It is well known that the parameter of K^trans^ characterized by wash-in rates is positively correlated with the capillary permeability in the tissue of interest (23). Because bevacizumab acts as an anti-tumor agent primarily by reducing the vascular permeability and blood flow, the K^trans^ values could be decreased along with the reduction in permeability (18). However, the steep decrease may aggravate local hypoxia, which may further lead to treatment resistance (39, 40). Meanwhile, bevacizumab may cause cell death and lysis and, thereby, reduce the cell density and the associated restrictive barriers of cell membranes. The destruction of the cell barrier perhaps leads to an increase in permeability, corresponding to an increase in K^trans^. On the other hand, it has been suggested that bevacizumab may transiently normalize tumor vasculature by pruning the immature and inefficient vessels and remodeling the remaining vessels, which would reduce the leakage of contrast agent (41–46). Therefore, the relationship between K^trans^ and OS may be related to the initial vascular status at week 3. With more normal blood vessels present, there may be fewer targets for vascular normalization during antiangiogenic therapy. Accordingly, the decrease in K^trans^ value would be smaller. Furthermore, the relationship between maintenance of the normal tumor vasculature and prognosis also has been reported in multiple studies (47, 48). Hence, the ultimate trend indicates that a small decrease in k^trans^ may be sufficient to identify high-risk individuals who would likely achieve improved OS with antiangiogenic therapy.

Recently, immunocheckpoint inhibitors represented by PD⁃1 have been widely studied in glioblastoma. Series of phase III clinical trials displayed disappointing results of PD⁃1 in glioblastoma, including the OS of recurrent glioblastoma in CheckMate-143, OS of newly diagnosed MGMT⁃unmethylated glioblastoma, PFS of newly diagnosed MGMT⁃methylated glioblastoma (49–51). In addition, it’s also worth to note that the efficacy of neoadjuvant was better than the adjuvant PD⁃1 therapy in recurrent glioblastoma (52). Although another study of the combination of neoadjuvant and adjuvant PD⁃1 therapy showed no survival benefit from neoadjuvant therapy in patients with resectable glioblastoma (53), both studies revealed that the neoadjuvant therapy was capable of regulating tumor immune microenvironment. These results indicated the importance of appropriate administration timing, combination therapy and tumor microenvironment status during immunotherapy. At the same time, potential biomarkers to predict the efficacy of immunotherapy maybe more conducive to individualized treatment. Researchers revealed that PTEN mutations, MAPK pathway alterations (PTPN11, BRAF) was correlated with anti⁃PD⁃1 immunotherapy (54). Since the activation of immune cells promotes normalization of tumor vessels, and RGD-based PET and DCE-MRI was potential to monitor the changes of tumor microenvironment by characterizing the intracranial neovascularization during immunotherapy (55).

Overall, the independent predictive roles of SUV_mean_ and K^trans^ may be related to their ability to represent the status of the tumor vasculature within the whole tumor, avoiding the influence of local heterogeneity. We measured mean values of permeability parameters (K^trans^, Kep, Ve, and Vp) instead of maximal or minimal values, which may also be useful to overcome heterogeneity. Based on their different predictive values for prognosis, the two imaging modalities evaluated may provide complementary rather than similar information regarding the prognosis of patients treated with antiangiogenic therapy in this study.

To the best of our knowledge, this is the comprehensive prospective registered clinical trial in which possible prognostic imaging biomarkers were detected. These encouraging results indicate that accurate, image-guided individual therapy is possible, and further research is warranted. Our clinical trial was a small sample exploratory investigation, and thus, larger studies with data for more molecular markers are needed to validate our findings.

## Conclusion

The present study shows that ^18^F-RGD PET/CT and DCE-MRI examination maybe useful for identifying glioblastoma patients likely to benefit from treatment with the combination of bevacizumab and CCRT, with a greater decrease in SUV_mean_ predicting better PFS as well as a smaller decrease in K^trans^ predicting improved OS.

## Data Availability Statement

The raw data supporting the conclusions of this article will be made available by the authors, without undue reservation.

## Ethics Statement

The studies involving human participants were reviewed and approved by Shandong Cancer Hospital and Institute. The patients/participants provided their written informed consent to participate in this study. Written informed consent was obtained from the individual(s) for the publication of any potentially identifiable images or data included in this article.

## Author Contributions

Study design: SY and JY; Data acquisition and analysis: LL, NL, LX, and YL; Patient sample collection: HZ, RT, SZ, ZC, and ZF. All authors have read and agreed to the published version of the manuscript.

## Funding

This study was partially funded by Natural Science Foundation of China (NSFC81872475, and NSFC82073345), Shandong Key Research and Development Plan (2017CXGC1209) to SY as well as Research Unit of Radiation Oncology, Chinese Academy of Medical Sciences (2019RU071) and the Academic Promotion Program of Shandong First Medical University (2019ZL002), the foundation of National Natural Science Foundation of China (81627901, 81972863 and 82030082), the foundation of Natural Science Foundation of Shandong ZR201911040452). to JY Tasly Diyi Ltd. provided the drug Temozolomide (DIQING). Roche Ltd. provided the drug Bevacizumab and part of funding. Tasly Diyi Ltd. and Roche Ltd. played no role in study design, data collection, data analysis and data interpretation.

## Conflict of Interest

The authors declare that the research was conducted in the absence of any commercial or financial relationships that could be construed as a potential conflict of interest.

## Publisher’s Note

All claims expressed in this article are solely those of the authors and do not necessarily represent those of their affiliated organizations, or those of the publisher, the editors and the reviewers. Any product that may be evaluated in this article, or claim that may be made by its manufacturer, is not guaranteed or endorsed by the publisher.

## Abbreviations

CBTRUS, Central Brain Tumor Registry of the United States; CNS, central nervous system; VEGF, vascular endothelial growth factor; VEGFR, VEGF receptor; PFS, progression-free survival; OS, overall survival; CCRT, conventional concurrent radiotherapy and temozolomide; MGMT, O6-methylguanine-DNA methyltransferase; EGFR, epidermal growth factor receptor; BBB, blood-brain barrier; RGD, arginine-glycine–aspartic acid; ^18^F-RGD PET/CT, ^18^F-AlF-NOTA-PRGD2 positron emission tomography/computed tomography; K^trans^, the volume transfer constant; DCE-MRI, dynamic contrast-enhanced magnetic resonance imaging; WHO, World Health Organization; KPS, karnofsky performance status; IMRT, intensity-modulated radiotherapy; 3D-CRT, 3D-conformal radiotherapy; FLAIR, fluid-attenuated inversion recovery; TR, repetition time; TE, echo time; NEX, number of incentives; FOV, field of view; Gd-DTPA, Gd-diethylenetriamine pentaacetic acid-bismethylamide; PCR, polymerase chain reaction; IDH, exon 4 of isocitrate dehydrogenase; ROIs, regions of interests; SUV_max_ and SUV_mean,_ maximum and mean uptake values; AIF, arterial input function; VOL, volume of lesions,; RANO, response assessment in Neuro-Oncology.
